# The HIV-1 transmission bottleneck

**DOI:** 10.1186/s12977-017-0343-8

**Published:** 2017-03-23

**Authors:** Samuel Mundia Kariuki, Philippe Selhorst, Kevin K. Ariën, Jeffrey R. Dorfman

**Affiliations:** 1 Division of Immunology, Department of Pathology, Falmouth 3.25, University of Cape Town, Anzio Rd, Observatory, Cape Town, 7925 South Africa; 2 Division of Medical Virology, Department of Pathology, University of Cape Town, Cape Town, South Africa; 3 International Centre for Genetic Engineering and Biotechnology, Cape Town, South Africa; 4 Department of Biological Sciences, University of Eldoret, Eldoret, Kenya; 5 Virology Unit, Department of Biomedical Sciences, Institute of Tropical Medicine, Antwerp, Belgium; 6 Department of Biomedical Sciences, University of Antwerp, Antwerp, Belgium

**Keywords:** HIV-1, Transmission, Bottleneck, Genital mucosa, Intravenous drug user

## Abstract

It is well established that most new systemic infections of HIV-1 can be traced back to one or a limited number of founder viruses. Usually, these founders are more closely related to minor HIV-1 populations in the blood of the presumed donor than to more abundant lineages. This has led to the widely accepted idea that transmission selects for viral characteristics that facilitate crossing the mucosal barrier of the recipient’s genital tract, although the specific selective forces or advantages are not completely defined. However, there are other steps along the way to becoming a founder virus at which selection may occur. These steps include the transition from the donor’s general circulation to the genital tract compartment, survival within the transmission fluid, and establishment of a nascent stable local infection in the recipient’s genital tract. Finally, there is the possibility that important narrowing events may also occur during establishment of systemic infection. This is suggested by the surprising observation that the number of founder viruses detected after transmission in intravenous drug users is also limited. Although some of these steps may be heavily selective, others may result mostly in a stochastic narrowing of the available founder pool. Collectively, they shape the initial infection in each recipient.

## Background

It has long been understood that the HIV-1 of the donor often exhibits a reduced viral diversity following transmission to a new host [1, 2]. More recently, about ten years ago, it became clear that this narrowing is usually very sharp, with only one or a very few viruses establishing a disseminated infection in the newly infected individual despite the high diversity of HIV-1 populations in most donors [3–8]. This phenomenon has become termed the “transmission bottleneck” of HIV-1 and is incompletely understood. Key questions about the precise nature of the selective events that result in the observed bottleneck remain unanswered.

The genetic bottleneck stems from physical and immunological conditions that prevent most variants within the incoming viral populations from establishing infection in a new host [9] and is reflected by the low efficiency of HIV-1 transmission from a single sexual exposure [4]. Thus successful infections in a new host frequently results from the dissemination of only a single variant after sexual transmission, in approximately 80% of heterosexual transmissions [6, 8], approximately 75% of transmissions in men who have sex with men (MSM) [10–12], approximately 70% of transmissions in mother-to-child [13] and 40–80% of transmissions in intravenous drug users (IVDU) [12, 14, 15].

Here we review the multiple steps at which selection potentially occurs, from donor compartments to established infection in the recipient. We look in the context of transmitted/founder (T/F) or acute virus genotypic and phenotypic traits identified and reported to date. Although there may be steps that result mostly in a stochastic narrowing of the available founder pool, some of the steps are likely to be heavily selective. This is suggested by the fact that the recipient’s disseminated infection is almost always derived from a minor variant within the diverse quasispecies of the donor [3, 4]. Increased risk of breakthrough of multiple variants after sexual transmission is mostly associated with factors that both compromise the genital mucosa and attract HIV-1 target cells e.g. genital ulceration and sexually transmitted infection (STI) [5]. This suggests that damage of the physical barrier of the mucosal surface and/or recruitment of target cells for HIV-1 infection increase the risk of subsequent systemic infection.

**Fig. 1:**
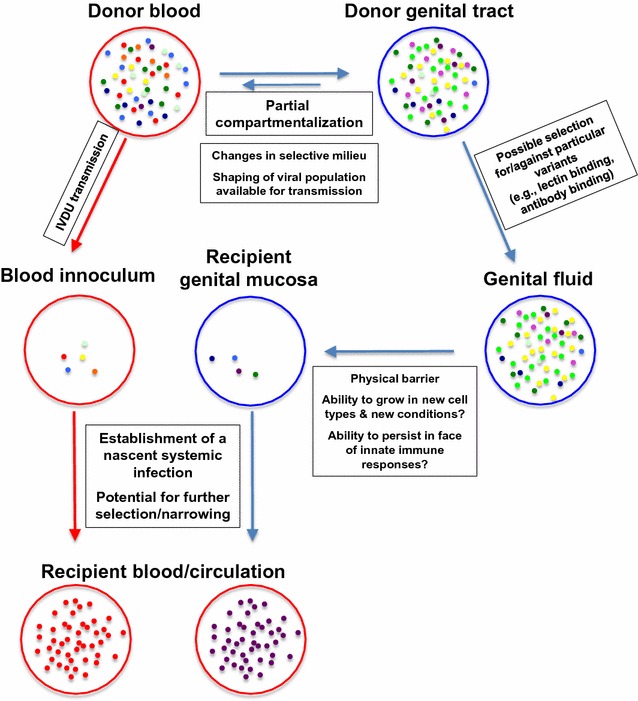
A schematic of some of the steps of sexual transmission and IVDU transmission of HIV-1 in which selective or stochastic narrowing of the HIV-1 population is possible. *Blue circles* genital tract; *blue arrows* steps of sexual transmission; *Red circles* blood/general circulation; *red arrow* transmission from parenteral exposure, e.g. IVDU exposure

Importantly, there is also evidence suggesting that the HIV-1 populations compartmentalize between general circulation and genital tract [16–21], and factors in semen [22] could select for or against particular HIV-1 traits. Thus, part of the bottleneck effect may already have occurred before transmission to a new donor. Additionally, the high rate of single variant populations early in infection in IVDU [12, 14, 15] may reflect substantial selection during the establishment of new systemic infections, after the HIV-1 has crossed the mucosal barrier. Thus, the HIV-1 transmission bottleneck may collectively describe events along every stage during the transmission process from the donor’s blood, all the way through to establishment of a stable, disseminated infection in the recipient. This is shown in schematic form in Fig. 1.

## Potential intra-donor selection during the transition from systemic circulation to the genital tract

Originally, it was thought that the virions and infected cells found in semen are directly imported from the blood [23]. However various studies have now shown that the genital tract constitutes a distinct viral compartment that locally produces viral particles and infected cells presumably under a different selective milieu than in the general circulation [24–27]. As a result, the viral quasispecies in the genital compartment are related to, yet distinct from that in blood. Therefore, during a transmission event, the viruses to which the recipient is initially exposed may already differ from the viruses found in the blood of the donor. Most data compare viral populations in semen to those in the blood circulation; however, limited data exist to suggest the possibility of a similar effect in the female genital tract compared to the blood circulation of the same donor [17].

Although the viruses in the genital compartment are thought to move back and forth between the blood and the genital compartment [20, 28, 29], generally this movement appears limited and doesn’t seem to negate the reduced genetic diversity observed in the genital tract [18]. Individual infected CD4^+^ cells or virions from the blood may infiltrate into pockets of uninfected target cells in the genital tract [30] to generate local foci of infection or even sustained, autonomous virus replication which would lead to clonal amplification or full compartmentalization of virus in the genital tract [18]. Studies of the male genital tract in macaques and humans indeed demonstrated that SIV and HIV-1 can replicate in leukocytes within the testes, epididymis, prostate and seminal vesicles during all stages of infection [31, 32]. These leukocytes, mainly T lymphocytes and to a lesser extent macrophages, are localized in the stroma and secretory epithelium of these organs. Infection of these cells could lead to the release of free viral particles and infected cells in the lumen and thus in the seminal plasma during ejaculation [33]. Prostate and seminal vesicles are likely the main source of cell-free HIV-1 in semen, as they display higher levels of infection than the epididymis and the testes [32]. This is supported by the fact that vasectomy has little or no effect on seminal viral loads [30, 34].

## Factors that may influence the transmission bottleneck in genital fluid

The genital fluid includes semen in males and cervical vaginal fluid in females. Genital fluids are known to contain proteins that can enhance or reduce the viral infectivity. In semen for example, a well-known enhancer of viral infectivity is the semen-derived enhancer of virus infectivity (SEVI) [22, 35]. SEVI is made up of peptides found in semen that aggregate into amyloid fibrils and are capable of enhancing virus attachment to target cells and increase infectivity by up to 400,000-fold [22] using a mechanism that involves cationic charges of the fibrils [36]. Studies are underway to determine whether the same viral enhancement happens in vivo.

Looking at pre-infection in women, inflammatory cytokines have been shown to enhance HIV-1 acquisition [37, 38]. Furthermore, it has also recently been shown that high levels of inflammation may select for transmission of viruses that are less infectious [39]. These effects likely reflect an impact of inflammatory cytokines upon the transmission bottleneck.

In HIV-infected men, transmission fluid contains both cell-free virus from the seminal plasma and cell-associated virus from seminal cells. The latter are usually the most abundant HIV-susceptible host cell in semen as seminal CD4^+^ T lymphocytes are often depleted during chronic infection [18]. The relative contribution of cell-associated vs cell-free seminal virus towards transmission is yet to be resolved. One study supports the transmission of cell-free virus as opposed to cell-associated virus [40], although this interpretation has been questioned [41]. Interestingly, viral variants isolated from seminal leukocytes are sometimes phylogenetically distinct from cell-free virions found in the seminal plasma [21] indicating that they may differ phenotypically and in their transmission potential. A discrepancy is often observed between the number of infected leukocytes and the cell-free viral load in semen [42]. It appears therefore that the HIV-infected cells in semen are not the primary source of cell-free virus in seminal plasma and that probably distinct sources contribute to either cell-free or cell-associated HIV-1 shedding in semen. Ordinarily, it is expected that cell-associated virus would represent older partially archived, more stable viral populations while the cell-free viruses in plasma would represent recently produced viruses, based upon the very short half-life of cell-free virus in blood or transmission fluids [43]. One would expect then that if the majority of transmitted viruses originated from infected seminal cells, that these would resemble earlier viruses in the donor. While this has been reported in a study by Redd et al. [44], the inferred donor ancestral virus identified in the recipient was shown to originate from blood but there was no analysis of virus from the genital compartments to trace as best as possible the steps from blood to transmission.

## Likely bottleneck while crossing the recipient mucosal barrier

The mucosa of the vagina consists of a multilayered, stratified squamous epithelium made up of four zones; basal, squamous, granular and cornified layers [45]. As this mucosa develops from the basal layers, cells become more flat and keratinized which restrict passive diffusion of materials through it, including HIV-1 [46]. The ectocervix has a similar profile but the endocervix is composed of a single layer of columnar epithelium covered with mucus. The mucus on these cells provides a protective barrier to infection, i.e. it forms a physical barrier in which virus particles can get trapped but also contains antiviral factors like secretory leukocyte protease inhibitor [47] and SDF-1 which is a natural ligand for CXCR4 [48]. The transformation zone that divides the endocervix and the ectocervix, has been suggested to be the most vulnerable region of the recipient for HIV-1 to gain entry. However, it has been shown that the virus can still establish a systemic infection when macaque blind vagina pouches after hysterectomy are exposed to cell-free virus [46]. Physical abrasion of the mucosal epithelium leads to an increased chance of infection. Presumably for this reason, the risk of infection through the anal sex route is approximately 10 times higher than via the vaginal route. This may be due to the relative fragility of the rectal mucosa, high risk of trauma during anal intercourse and/or more abundant HIV-1 target cells in the rectal mucosa. The established major sites of HIV-1 transmission in males are the inner foreskin, tissue directly underneath the foreskin, and the urethra [49]. Presence of HIV-1 target cells on the inner foreskin renders it susceptible to HIV-1 infection [50–52]. Compared to the systemic circulation, a substantially larger proportion of CD4^+^ T lymphocytes present in the foreskin express CCR5 coreceptors [53].

It is still not completely established to what extent an intact mucosal barrier selects for particular viral characteristics over and above acting merely as a physical barrier to entry of most viruses. HIV-1 uses several mechanisms to cross the mucosal barriers of the recipients. It has been suggested that transcytosis and intraepithelial transmigration of infected donor cells are the common mechanisms that the virus uses to access the submucosal tissues of the host. In addition to this, HIV-1 can infect intra-epithelial Langerhans’ cells [54, 55] and also possibly intra-epithelial CCR5^+^ CCR7^hi^ CD4^+^ T cells that can migrate in and out of the female genital tract and hence can disseminate infection [56].

## Establishment of an initial focus of infection in the recipient’s mucosa

Once HIV-1 manages to breach the mucosal barrier, it will reach submucosal target cells such as dendritic cells, macrophages, and CD4^+^ T lymphocytes [57]. Although macrophages and dendritic cells can sustain productive infection, resting CD4^+^ T lymphocytes (i.e. without markers of activation) are probably the first cells to be infected [58–60]. Studies in macaques have shown that small clusters of infected CD4^+^ T cells form in the mucosal tissue within 3–4 days after vaginal SIV exposure [9]. Not surprisingly, these initial foci of infection are consistently found in the endocervix and transformation zone [9], although infection in other regions of the female genital tract does occur [61].

Viral exposure immediately increases MIP3-α/CCL20 expression in the endocervical epithelium. This chemokine attracts plasmacytoid dendritic cells, which in turn recruit T cells and macrophages through MIP1-β [60]. Unfortunately this outside-in endocervical mucosal signaling system has the paradoxical adverse effect of recruiting new target cells to the site of infection. As a result, the innate immune response fuels the expansion of the initial founder population of infected cells, rather than preventing it [62]. Subsequently lymphatic drainage will spread this initial infection to the draining lymph nodes. Interstitial dendritic cells and possibly also macrophages are believed to facilitate this process [63]. DCs can trap HIV-1 on their cell surface through the expression of the C-type lectin DC-SIGN (specific ICAM3-grabbing non-integrin), which can bind gp120 [64]. Mannose receptors, which are expressed by macrophages, could play a similar role [65]. Like LCs, DCs are antigen presenting cells that migrate from the mucosal tissue to the lymph nodes to activate CD4^+^ and CD8^+^ lymphocytes. However, in contrast to langerin (CD207), binding of gp120 to DC-SIGN does not lead to HIV-1 degradation but rather preserves its infectivity for several days [63]. Virions are sequestered into surface-accessible compartments of the DCs arising from membrane invaginations [66]. Female to male transmission is less well studied; but, the initial foci of infection appear to occur most prominently in the inner foreskin in uncircumcised [67] and urethra in circumcised and uncircumcised men [68]. Finally, although initial foci of infection in the genital tract are associated with successful transmission, they may not be a prerequisite. It remains possible that, through a compromised epithelium or transmigration, migratory immune cells carrying HIV as described above, could bypass the submucosa and directly establish infection in the lymph nodes or GALT. This mechanism is thought to explain systemic infection in the presence of high local concentrations of topical antiretroviral drugs that inhibit replication in the genital tissue [69].

## The GALT and systemic dissemination

Upon arrival in the lymph nodes, HIV-1 exploits the immunological synapse that is normally formed between the antigen presenting DCs and naive CD4^+^ T cells, and transforms it into a virological synapse [70, 71]. Within this synapse, filopodial extensions emanating from CD4^+^ T lymphocytes make contact with the sequestered HIV-1 virions thereby enhancing the efficiency of HIV-1 transmission [71]. The establishment and expansion of the small founder population at the portal of entry and the subsequent dissemination to the draining lymph nodes will eventually result in a self-propagating infection, which will spread to the secondary lymphoid tissues such as the spleen and GALT. This marks the end of a 7–10 day period, also known as the eclipse phase, in which virus is not yet detectable in the blood [72]. From then on, the infection is systemically apparent, leading to substantial depletion of gut CD4^+^ T cells during the acute phase and immune activation in the early asymptomatic phase.

## Transmission bottleneck among intravenous drug users (IVDU)

There appears to be a substantial bottleneck during HIV-1 transmission among intravenous drug users (IVDUs). The disseminated infection seemed to be derived from a single variant in 40–80% of the transmissions of new infections that were analyzed [12, 14, 15]. In an analysis that combined data from 5 different studies, only one T/F virus was detectable in 21 of 32 (66%) recent intravenous drug use-associated HIV-1 infections [12]. In aggregate, this is not different from the rate of infections traced to a single T/F virus following sexual transmission [12]. This strongly suggests that a substantial bottleneck at or near transmission exists during IVDU-associated transmission, in which there is no mucosal barrier to transmission.

The data used in this 5-study analysis almost exclusively come from two research groups working in different settings. In a North American cohort, 4/10 (40%) of the IVDU infections studied were traced to a single T/F. Although this is significantly lower than the rate of single founder infections identified following sexual transmission [14], this study nonetheless suggests that there remains a substantial bottleneck in IVDU transmission despite the absence of a physical barrier to transmission. In a Russian IVDU cohort [15], 9/13 (69%) infections studied were traced to a single T/F. If this latter study is combined with unpublished analysis of 7 further infections from the same research group, then 16/20 (80%) of the IVDU infections studied were traced to a single T/F virus from this Russian cohort [12].

A substantial limitation to interpretation of these studies is that the size of the initial inoculum in IVDU cases is very difficult to estimate because, in part, it depends upon the volume of blood transferred during the use of shared needles and/or syringes, upon the infectiousness of the transferred virus and upon the donor’s viral load. The volume of blood drawn into the syringe to confirm that the needle is in a vein, will be highly variable (perhaps 100 μl) and subsequently diluted by a variable volume of drugs in the syringe. In addition, practices such as booting (i.e. drawing blood into the syringe a second time and re-injecting it to rinse residual drug solution out of the dead-space) as well as flushing the needles with water before sharing, will all affect the final volume of donor blood that is transmitted to the recipient. In simulations, high dead space syringes transfer approximately 84 μl without rinsing and 1 μl of donor blood with rinsing, and low dead space syringes transfer ~2 μl without rinsing and much smaller volumes (<0.001 μl) with rinsing [73, 74]. There is thus a wide range of possible transferred volumes, even under relatively controlled laboratory conditions.

A second factor that needs to be considered, will be the infectiousness of the HIV-1 in the retained donor blood. This will be affected by practices such as drug heating, the time between needle sharing, etc. Third, the size of the virus inoculum will be determined by the viral load of the donor. Viral loads of recently infected IVDUs [14, 15] and individuals infected by heterosexual transmission [75] are very often over 100,000 cp/ml, (100 cp/μl), in almost half of the study participants tested. Recently infected individuals may account for a disproportionately large fraction of HIV-1 transmission under many conditions [76]. In any event, a log mean viral load of 16,000 cp/ml was calculated for North America and Europe (where these IVDU studies were conducted) using a large dataset derived mostly from chronically infected individuals [77]. These viral loads, particularly those of recently infected individuals, are high enough that the presence of multiple variants in submicroliter volumes of blood is probably common. Despite this, new HIV-1 infections in IVDUs are very frequently traceable to a single founder virus, similar to what is observed for sexual transmission [12].

Another limitation to these studies is that it is not possible to rule out that some of the transmissions presumed to occur via the IVDU route could also have been actually transmitted sexually. In the case of the 20 transmissions analyzed from Russian IVDUs, the transmission would almost certainly need to have been from another drug user (even if sexually transmitted), as the IVDU epidemic in the region is predominantly subtype A and the sexually transmitted epidemic is predominantly subtype B [78]. Finally, it is also not possible to rule out transmission of more than one very closely related virus. This may be more likely when the donor has an acute infection that has not fully diversified.

Despite these limitations, when aggregating all available data, the overall rate of new infections traced to a single variant in presumed IVDU transmission appears to be much higher than what can be expected from the estimated viral inoculum, and statistically indistinguishable from the rate in sexual transmission [12]. That there is a drastic drop in viral diversity from intravenous inoculum to new HIV-1 infection is supported by a study in rhesus macaques [79]. Five macaques were challenged with an intravenous dose of 2 × 10^5^ viruses of one of two viral isolates with diversity typical of early chronic HIV-1 infection, plausibly a larger dose than is normally experienced during IVDU transmission. In three of the five macaques, the resulting infection was traced to between 1 and 4 distinct variants. Thus, even under these controlled conditions, the diversity of the HIV-1 infection from intravenous exposure is drastically reduced compared to the diversity found in the inoculum.

To the extent that these data are explained by selection following IVDU inoculation, we must conclude that the mucosa of the genital tract are not absolutely required to produce a transmission bottleneck. We suggest that there may be substantial selection and/or stochastic narrowing at the level of establishment of systemic infection (see Fig. 1).

If so, further thought must be applied to the role of the genital tract mucosa as a physical barrier to HIV-1 infection in sexual transmission. As already noted, analysis of an aggregate of currently available data suggest that the rate of new infections traceable to a single variant is not different between IVDU and sexual transmission [12]. However, there are substantial uncertainties associated with concluding that the mucosal barrier is not meaningful to HIV-1 transmission. In particular, there are studies that suggest that genital mucosal surfaces do provide a substantial barrier to HIV-1 viral particles [3, 4, 61, 80–85] that is likely to be meaningful to sexual transmission, although not essential for the transmission bottleneck. Nonetheless, in light of the substantial bottleneck associated with IVDU transmission, it is challenging to understand why sexual transmission to recipients with a range of STIs (such as syphilis and other ulcerating infections [86, 87], gonorrhea [88] and chlamydia [89]) leads to an increased risk of HIV-1 infection, and possibly an increased risk of infection by multiple HIV-1 variants [5, 90] (see below). Perhaps the effects of the STIs that actively promote the establishment of HIV-1 infection are the key, important effects rather than effects associated with compromise of the mucosal barrier.

## Sexually transmitted infections and risk of multivariant transmission

Several types of sexually transmitted infections (STIs) increase the risk of HIV-1 infection in a potential recipient [38, 91]. There is also evidence for an association between presence of STIs and initial infection with multiple HIV-1 variants [5, 90]. However, neither study identified associations with particular STIs; they only observed associations with any vaginal/urethral discharges in the recipients [5] or when grouping together occurrences of a number of STIs [90]. Other studies were unable to observe this association [92, 93]. It is possible that these latter studies were underpowered to detect the association; perhaps they failed to have a sufficient number of cases of the particular STIs most responsible for the observed increased risk of multivariant transmission. These effects could be mediated by breaks in the integrity of the mucosal barrier [94], inflammatory cytokines [39] and/or availability of more, better or more accessible target cells for HIV-1 [91], particularly cells capable of sustaining HIV-1 infection and migration to other tissues [54–56]. These possibilities may not necessarily be mutually exclusive. Because of the IVDU transmission evidence summarized above, we disfavor explanations associated with breaks in the integrity of the mucosal barrier.

## Traits reported to be different between transmitted/founder (T/F) vs chronic viruses

Clear evidence that the T/F virus is selected from a highly diverse population present in a chronically infected donor [7, 8] has sparked substantial efforts to elucidate the characteristics selected for in T/F virus. Several research groups working on characterization of the T/F virus have identified traits that are different between T/F viruses and chronic viruses. This includes genotypic characteristics such as levels of envelope glycosylation, length of envelope variable loops, being closer to an archived or consensus-like genotype, but also phenotypic characteristics such as CCR5 and CXCR4 utilization, neutralization sensitivity, increased infectivity and replication capacity, Type I interferon (IFN) resistance, and susceptibility to capture by α4β7.

### Transmission signature patterns in envelope sequences

Analysis of subtype B envelope sequences from acutely and chronically infected participants identified potential transmission signatures in the signal peptide, close to the CCR5 and CD4 binding sites and within the gp41 cytoplasmic tail and cytoplasmic domains [11]. Using the most stringent approach, they found two differences. Sequences from acute infection were more likely to carry a histidine at position 12, and were more likely to lack a potential N-linked glycosylation (PNG) site at position 415 than those from chronic infection. Position 12, within the signal peptide has been shown to be involved in trafficking of the nascent Env polypeptide to the endoplasmic reticulum and incorporation into viral particles and viral infectivity [95]. Changes in signal peptides have previously been shown to influence viral infectivity and Env expression [96]. Furthermore, sequences from chronically infected participants were more likely to carry threonine at position 415, resulting in the formation of a PNG site that was associated with sensitivity to neutralization [11]. It was suggested that the presence of a PNG site at position 413–415 was selected against in acute viruses because it might impact viral infectivity or it could play a role in the mechanism of transmission.

### Closer to archived or consensus-like genotype

A study analyzing the envelope sequences of viruses sampled from genital fluid and blood plasma of transmitting pairs, observed that the T/F virus sequences more closely resembled sequences from blood [17]. In addition, a study among Ugandan discordant couples showed that the transmitted virus sequences resembled those of ancestral variants, i.e. variants sampled earlier in time, as opposed to the contemporaneously circulating strains at the time of transmission in the donor [44]. This was attributed to sequestration of the virus in long-lived reservoir cells that were infected early in the course of infection, but subsequently maintained at low levels in circulation as latently infected cells [44]. This work is supported by Carlson et al. showing that there is a bias in recently transmitted viruses for consensus-like amino acids generated from Gag, Pol and Nef proteins in 137 epidemiologically-linked transmitting pairs infected with subtype C viruses [97]. In addition, using infectious molecular clones generated from six-linked heterosexual transmission pairs, it was shown that consensus-like genomes that were more sensitive to donor antibodies were selected for during transmission [98]. This group observed a selection bias towards consensus-like virus by measuring the pairwise distance of each variant to a database-calculated subtype C consensus. T/F variants had reduced pairwise distances to consensus as compared to non-transmitted variants [98]. This idea of transmission of earlier variants is supported by Love et al., which showed that the initial founder virus circulates at low levels, even long after transmission [99], suggesting that it or closely related viruses persist long into infection and may be available to transmit to new hosts. Taken together, the current understanding is that the T/F virus originates from a pool of variants that more closely resemble those that already existed before the transmission event than chronic viruses do.

### Potential N-glycosylation sites and loop lengths in Envelope

One important characteristic of the T/F virus is the differential glycosylation levels of envelope. The glycans are carbohydrates that make up more than 50% of the HIV-1 envelope molecular weight. Initially, the glycans were thought to primarily influence immune escape, and are selected to shield antibody recognition site(s) [100]. However with subsequent studies, it became more and more clear that the extent of glycosylation has an influence on viral transmission and also involve structural characteristics. For example, they are part of the epitope of some broadly neutralizing antibodies [101–104]. Derdeyn et al. [105] first suggested that transmitted viruses had fewer N-linked glycan sites because they observed that these viruses often had changes in the length of the variable loops. Similarly, it has been reported that T/F viruses from subtypes A, C and D carry more compact envelopes with fewer N-linked glycosylation sites [105–107]. The reduced glycosylation of the T/F virus has been linked to enhancement of binding of the virus to the α4β7 integrin which is a homing marker for CD4^+^ T cells to the GALT [108]. However, this trend for fewer PNG sites is not consistent across all studies. It has not been observed in subtype B [107, 109–111], suggesting that phenotypes associated with one subtype or transmission route/conditions might not hold true for another. After transmission, the viral populations that subsequently evolve over the course of infection accumulate additional PNG sites [112]. A study done in a large cohort of subtype C non-linked acute and chronic viruses by Ping et al. [106] showed a 5% overall difference in total glycosylation count between the acute and chronic viruses. Although this difference may appear small, it may reflect a much larger difference in a key subset of PNG sites that are relevant for increased transmission capacity. These changes may reflect subtle differences in the optimal structural characteristics of Env in T/F viruses vs the optimal structural characteristics of Env in chronic viruses. This could include interactions with α4β7 integrin or other receptors on distinct cell types, or type I interferon resistance (see below). They also plausibly reflect a lesser need to evade antibodies very early when infection is first established.

The HIV-1 envelope gp120 has five hypervariable subregions V1–V5 that are separated along the amino acid sequence by five constant regions C1–C5 and interconnected by cysteine residues. They participate in several important functions in the evolution of the virus in the host. The V3 variable loop is the main determinant of coreceptor usage while the V1–V2 region participates substantially in masking the host neutralizing antibody target sites. These properties are influenced by the sequence length of the envelope, the changes in glycosylation pattern and changes in amino acid sequences [113]. The T/F virus was found to have shorter variable regions as compared to their chronic counterparts [105, 112–116] meaning that these viruses have more compact Env glycoproteins and there is a possibility they could be interacting more efficiently with the target cells in the genital mucosa [5, 107, 117, 118]. Additionally, during early infection variants with shorter V1–V2 regions are seen to have a competitive growth advantage over the others. These variable loops then increase in length as the virus goes through chronic infection and then decline once again in late stage infection. The change in length especially of the V1–V2 regions is due to deletions, insertions and many substitutions that appear to reflect escape from immune response of the host [114]. At the population level, during the HIV-1 epidemics it has been reported that the V1–V2 region has grown in length making the current viruses have moderately longer V1–V2 [112, 114, 119] and more glycosylation sites [120] than historically older viruses.

Despite attempts with large data sets [97, 106], few strong associations between particular PNG sites or patterns of distinct PNG sites have been reported that are more frequent or less frequent in T/F isolates compared to chronic isolates, and those that are reported are fraught with complex associations between the presence of different PNG sites (JRD, unpublished data). This suggests a complexity that is poorly understood. For example, the selective processes may affect particular PNG sites differently in different contexts, i.e. in different isolates or different transmission modes or conditions. Also, different antibody responses may select for or against different PNG sites in different individuals, complicating detection of otherwise simple associations. More analyses with larger and more carefully matched sequence sets may reveal further insight into the role of particular PNG sites in selection of T/F viruses.

### CCR5 and CXCR4 utilization

Of all the phenotypic features of the T/F virus, CCR5 utilization seems to be the most consistent. If specific viral sequences are selected for during transmission one would also then expect that the transmitted virus would favor interaction with specific cellular receptors and co-receptors. Several research teams have shown that CCR5 utilization is a key phenotype of the early transmitted virus [121, 122]. Keele et al. [8] showed that 98% of T/F viruses were CCR5 tropic. It was previously suggested that the CCR5 tropism of transmitted variants was due to the availability of macrophages at the site of transmission. However, the apparent dependence upon high CD4 levels, and the similar macrophage infection capacities observed in chronic and T/F viruses, suggested that macrophages were unlikely to be the primary target cell during transmission [111, 123]. Nonetheless, others show primary infection of urethral macrophages, suggesting that the need for high levels of CD4 is not absolute [68].

Etemad et al. [124] took the V1–V5 region from early and chronic variants and cloned them into an isogenic HIV-1 backbone to generate chimeric infectious molecular clones (IMCs), the chronic infection variants replicated to higher titers in cells with lower CCR5 levels compared to those from early infection, suggesting that T/F viruses might require higher levels of CCR5 for infection. However when Wilen et al. [125] compared the envelopes from 24 T/F viruses to those of 17 from chronic infection in a pseudovirus assay for their ability to utilize CCR5, CD4^+^ T cells subset cell tropism, fusion kinetics and dendritic cell trans-infection, they only found that the transmitted variants were marginally more sensitive to CD4 binding site antibodies than those from chronic infection. Comparing viral envelope proteins from acute and chronic infections (all subtype C), Ping et al. [106] reported that chronic variants were able to use both a maraviroc-sensitive and an alternative maraviroc-insensitive conformation of CCR5, confirming earlier work [126]. Overall, these conflicting results could suggest that CD4 and CCR5 levels are not important for HIV-1 transmission. However, effects from the viral model used (i.e. pseudoviruses vs IMC’s), the study of different subtypes in different studies, and use of unmatched T/F and chronic samples (i.e. from different participants) may be part of what complicates drawing a firm conclusion.

### Neutralization sensitivity

Autologous neutralizing antibodies are usually detectable only after the first few months of HIV-1 infection [100, 127–129, 130]. Nonetheless, there is evidence that antibody-based selection sometimes exerts selective effects soon after transmission and before the neutralizing antibodies were detectable by pseudovirus-based assays [131]. The selective pressure of antibody persists for years in the chronic phase of HIV-1 infection [132]. Escape and production of new antibodies, from which the virus escapes again, occurs in iterative cycles [129, 133–135]. These cycles are also observed in experimental macaque models of HIV-1 infection [136].

Evidence from B cell depleted macaques suggests that the constant antibody pressure gives some protection to the host, including reduced viral load [137, 138] and protection from disease progression [138]. A third study showed no protection; however, the sham-depleted controls did not develop detectable neutralizing response against the challenge virus or these responses were severely delayed [139]. In all cases, interpretation was complicated by the fact that complete depletions seem to be rarely achieved in macaques [137–139]. In any event, it seems clear that any protective effect of antibody is dwarfed by a substantial protective effect of CD8^+^ T cell responses [140]. Depletion of B cells in African green monkeys had no effect upon drops in CD4^+^T cells or (generally rare) progression to disease [141]. However, even CD8^+^ T cell depletion has little effect [142], despite overwhelming evidence for their importance in human HIV-1 infection. This, along with the rarity of progression to disease [143] and different dynamics of infection [144, 145] in SIV-infected African green monkeys calls into question to what extent they are a useful model for HIV-1 infection in humans. Interestingly, in human HIV-1 infection, B cell depletion of an individual resulted in the temporary appearance of a neutralization-sensitive variant associated with a higher viral load set point [146], suggesting a role for antibody in protection of humans.

Nonetheless, it is clear that presumably higher antibody pressure from more broadly neutralizing antibody responses does not result in protection from HIV-1 disease. Time to disease progression was not longer in individuals with more broadly neutralizing antibody responses [147, 148]. Clearly, the levels and breadth of neutralizing antibodies achieved in human populations was not sufficient to force selection of HIV-1 variants that were less fit or otherwise less able to induce disease. There is some evidence that such breadth and potency is experimentally achievable [149, 150], although presumably higher than normally achieved during natural infection.

Although broadly neutralizing antibodies do not confer protection on a human population level, neutralizing antibodies impose substantial selective pressure upon viral populations. It is possible that such pressure makes chronic viruses overall more resistant to antibody, particularly neutralizing antibody, than T/F viruses. There are two related, but separable measures of neutralization resistance: (1) resistance to autologous antibody (of the donor in a transmission pair) or (2) overall resistance, usually measured with pools of serum or blood plasma [151, 152].

Viruses establishing new infections through the heterosexual route were sensitive to antibodies obtained from the infecting donor [98, 105], but not more sensitive to a set of heterologous blood samples [153]. This sensitivity to donor antibodies was more apparent for subtype C virus [105] and not in a subtype B MSM cohort [110] where only 2/8 from the chronically infected source partner showed increased neutralization sensitivity to autologous neutralization [110]. The reason for the discrepancy between the studies is not clear; the infecting clade and the primary mode of transmission were both different. Nonetheless, the subtype C results raise the possibility that a shift in selective milieu over transmission favored new founder isolates that were less resistant to the neutralizing antibodies of the donor.

There is evidence to suggest that antibody can inhibit superinfection if the recipient has high titer neutralizing antibody directed against the superinfecting isolate. Two studies [75, 154] identified several superinfection pairs and showed that the recipient’s serum generally contained little neutralizing activity directed against the superinfecting viral population (usually ID_50_ < 100). The superinfecting variants were not more sensitive to a heterologous serum pool than viral isolates from the pre-superinfection lineage, suggesting that they were not overall more neutralization sensitive [154]. Delay in development of neutralizing antibody responses and a lower magnitude of neutralizing activity were noted prior to superinfection in the superinfected recipients, when compared to recipients that were never detectably superinfected. This suggests that more robust neutralizing responses may have been protective [75]. It has been difficult to show stronger evidence that antibody can inhibit superinfection. This is because it has not been possible to compare to superinfecting viruses that failed to establish themselves. However, in the age of large deep sequencing projects, it is becoming possible to detect these failed superinfections [155]. Taken together with direct evidence for superinfections that grow and eventually fail [155], these data strongly suggest that particular isolates can be efficiently selected against by antibodies that neutralize them, and that isolates from chronic infection may have been iteratively selected to be neutralization resistant.

### Replication capacity and infectivity

HIV-1 establishes initial foci of infection in the genital mucosa. It was therefore hypothesized that viruses with enhanced infectivity and/or replication capacity will be preferentially transmitted. Parrish et al. [156] compared infectious molecular clones derived from chronic and acute infection and found that viruses from acute infection were on average 2 times more infectious yet did not find a difference in replication capacity. A potential confounder of this study was that these viruses were not derived from epidemiologically linked transmission pairs. Two subsequent studies, that used matched donor-recipient pairs, failed to show that transmission selects for viruses with increased infectivity and/or replication capacity [98, 157]. Both studies characterized only a limited number of donor and recipient viruses. Selecting only 1–8 viral variants per transmitting donor and 1 variant per recipient might not have sufficient statistical power to reveal differences in infectivity or replicative capacity. Oberle et al. [157] measured replicative capacity using bulk viral isolates from in vitro PBMC culture, which should represent part of the in vivo viral quasispecies. Nonetheless, this might have selected for the outgrowth of viral variants fit for growth in culture (rather than in vivo persistence in chronic infection or during transmission) or outgrowth of archived viruses that no longer represent the plasma virus at that particular time point. (See the next section below for further discussion of potential technical differences between these reports.) Oberle et al. wrote that their results “support(ed) the notion that, at least in subtype B infection HIV-1 transmission is to a considerable extent stochastic,” which has generated some discussion [158, 159].

A recent study by Iyer and co-authors [160] circumvented these problems by generating 300 single genome viral isolates using limiting dilution from transmission pairs. Isolates were from plasma and genital secretions of chronically infected donors and from plasma of their matched recipients. Iyer et al. were thus able to plot the distribution of infectivities and replication capacities in the quasispecies of each donor and recipient pair. They convincingly show that donor plasma isolates exhibit a wide range of infectivities and replication capacities. Importantly, transmitted viruses are on average 3 times more infectious and replicate 1.2–1.7 times more efficiently than the non-transmitted donor viruses. Interestingly, overall, viruses derived from genital secretions did not show a statistically significant increase in replication capacity or infectivity as compared to their corresponding donor plasma isolates, although a trend towards modestly increased infectivity could be seen. These data suggest that the recipient’s genital mucosa (or perhaps the subsequent establishment of the nascent systemic infection) selects for viruses with enhanced replication capacity.

### Type 1 interferon sensitivity

Selection processes operating during transmission may favor viral variants able to resist attack from host innate immune responses found in the genital tract, such as type I interferons. It has been shown that T/F viral isolates appear more resistant to IFN-α than viruses derived from chronic infection in some [156, 161] but not all studies [98, 157]. Plasmacytoid dendritic cells are rapidly recruited to new foci of SIV infection in the endocervical mucosa in macaques and secrete high levels of type I interferons and interferon-stimulated genes in the endocervical mucosa [62], which could be expected to limit viral replication or select for traits that promote interferon resistance. Additionally, topical application of IFN-β in rhesus macaques provided protection from infection [162], suggesting that the presence of type I interferons can induce a state in the female genital tract that protects against infection. Thus, it is plausible that part of the selection around the time of transmission may involve selection for growth in the presence of substantial levels of type I interferons in the genital tract. In contrast, during chronic infection, it is possible that the virus may have the opportunity to grow under conditions or at anatomical sites in which type I interferons exert less of an effect upon viral population growth.

In addition to the assessments of replicative capacity described above, Iyer et al. [160] measured the inhibition of IFNα2 and IFNβ upon primary HIV-1 isolates from transmission pairs. The interferon resistance of donor plasma-derived viruses varied over a wide range. In comparison, isolates from recently infected recipients were uniformly resistant to type I interferons, and were, on average, 7.8-fold and 39-fold more resistant to inhibition by IFNα2 and IFNβ, respectively, compared to donor isolates. A limited proportion of this selection may have occurred at the transition between donor circulation (blood) and the donor genital tract, as suggested by a 2.5-fold increase in IFNβ resistance in genital tract isolates. Presumably, the remainder of the transition occurs through the process of crossing the recipient genital mucosa, replicating there, and then establishing the new disseminated infection. This is in line with earlier results suggesting increased interferon resistance of recipient viruses compared to later chronic isolates from the same individuals [161, 163]. In contrast, Deymier et al. [98] did not observe any effect upon IFNα resistance and Oberle et al. [157] found recipient viruses to be modestly *more* sensitive to IFNα than their paired transmitter viruses. As noted above, these studies may have been limited in the number of viruses evaluated [98], or in the type of virus isolated [157], which could have impacted the results. In addition, both studies measured IFNα resistance in response to a single dose of IFNα which may be less accurate than determining the IC_50_, as done by Iyer et al. [160]. Importantly, IFNα resistance is not necessarily constantly low during chronic infection, but increases as patients approach progression to AIDS disease [164, 165]; therefore, the difference in IFNα resistance may be sensitive to when during the course of infection a virus is transmitted. Finally, the mode of transmission might play a role, with Iyer et al. (4/7 transmissions were female-to-male) evaluating more stringent and potentially more selective bottlenecks [97] as opposed to Oberle et al. (mainly MSM) [157]. This would not explain the discrepancy in conclusions with Deymier et al. (5/6 transmissions were female-to-male).

### Virus release from infected cells and the role of *vpu*

Transmitted founder viruses were shown to exhibit higher virion release capacity than chronic control viruses [166]. This was confirmed in a much larger study on epidemiologically-linked transmission pairs showing that recipient isolates had 4.2-fold higher odds of virus release from infected cells than donor isolates. Interestingly, donor viruses which were isolated in the presence of high levels of IFNs, also showed enhanced virus release as compared to untreated donor isolates [160]. These data suggest that the production of cell-free virus is a determinant of IFN resistance, which, as described above, is important to overcome the innate immune response.

Virus release from infected cells is known to be restricted by the cellular restriction factor tetherin which is a protein-based tether that can retain HIV-1 virions on the cell surface [167]. HIV-1 has evolved Vpu proteins to counteract tetherin to significantly enhance virus replication and release in human CD4^+^ T cells particularly in the presence of IFNs [167]. Nonetheless, even in the absence of Vpu, T/F viruses still produce more cell-free viruses [166]. Moreover, in a study to determine the importance of Vpu activity in HIV-1 transmission, no difference was found in the functionality of Vpu from T/F isolates as compared to isolates from chronic infection [168]. This suggests that Vpu is not primarily responsible for the observed difference in HIV-1 particle release and that other factors presumably play a role.

### Role of α4β7 integrin and gut-associated lymphoid tissues (GALT) in HIV-1 selection

A natural mechanism exists whereby white blood cells move from the genital tract to the gut associated lymphoid tissue (GALT) [169], which is a primary site of HIV-1 replication [170]. HIV-1 populations can use this mechanism to escape the genital tract. This is made possible by the ability of HIV-1 envelope to bind α4β7, an integrin homing receptor on the surface of activated CD4^+^ T cells [171], including those which are present at the site of sexual transmission [172] This happens within days of infection. The virus is transported to the Peyer’s patches and mesenteric lymph nodes where massive HIV-1 replication takes place [171, 173, 174]. Viruses with greater α4β7 binding capacity may therefore be selected for expansion in the gut. The selection in GALT has the potential to explain unexpectedly high frequencies of single variant transmission reported in intravenous drug users (IVDU) discussed above.

The ligands of α4β7 integrin bind to the HIV-1 gp120 subunit of Envelope through a tripeptide motif with a central aspartic acid [175]. A similar binding site is often found in V2 region of HIV-1 gp120 at position 179–181 (HXB2 numbering) with the aspartate at position 180 being conserved across 98% HIV-1 isolates [171]. The consequence of infection of the GALT by HIV-1 is rapid depletion of T cells. Specifically, the depletion of the Th17 subset have been implicated in deterioration of the intestinal mucosa integrity and therefore leading to translocation of microbes and microbial components in the systemic circulation [169, 176] leading to immune activation. Binding of the gp120 to the α4β7 integrin is not an essential requirement as is the binding to CD4 receptor or the CCR5 co-receptor but it is thought to be important only in the early stages of HIV-1 infection [171]. In fact, is has recently been shown in an acute infection cohort that the interaction with α4β7 varied between isolates and within individuals over time [177]. Nevertheless, HIV-1 target cells with high concentration of α4β7 has been shown to be more susceptible to HIV-1 infection, a factor that may also be attributable to the high levels of CCR5 found in these cells as well [178]. In addition, preferential infection of α4β7 expressing leukocytes would increase the chances of HIV-1 to be disseminated to the GALT. This may be a discrete trait; Nawaz et al. [108] showed that shorter C3/V4 were associated with efficient binding of HIV-1 to α4β7. A recent study has shown that challenging macaques intravenously with anti α4β7 antibodies protects them from a repeated dose challenge with SIV_mac251_ infection due to their GALT being protected from infection [179–181]. In sum, α4β7 is used as an attachment factor that lowers the entropic barrier for binding of HIV-1 to CD4 and CCR5 which overall gives a selective advantage to the virus during the early stages of infection [177]. Some in vitro studies have failed to confirm the contribution of this integrin in HIV-1 infection [98, 163, 182, 183]. Nonetheless, much of the available evidence suggests that viruses with greater α4β7 binding capacity may be selected for expansion in the gut. Selection in GALT has the potential to explain unexpectedly high frequencies of single variant transmission reported in intravenous drug users (IVDU) discussed above.

### Macrophage tropism

Unlike CD4^+^ T cells, macrophages have low levels of surface CD4 and have mostly been observed in the central nervous system of infected patients [184, 185], late in disease [186] and more recently in the male genital compartment [187]. Probably the most well studied compartment as far as macrophage tropism is the central nervous system which has 4 types of macrophages; microglia, meningeal macrophages, perivascular macrophages and macrophages of the choroid-plexus [188]. The macrophage-tropic viruses use the CCR5 coreceptor for entry [189] and are more sensitive to inhibition by soluble CD4 and some mAb targeting the V1/V2 region [190]. The nature of the transmitted virus has also been studied in the context of macrophage tropism. Most of these in vitro studies have been done by infecting monocyte-derived macrophages (MDMs) using HIV-1 isolates, infectious molecular clones (IMCs) or pseudotyped viruses. More recently, some studies have used CD4 Affinofile cells expressing low amounts of CD4 receptors as a surrogate for macrophage tropism [184]. The advantage of Affinofile cells is that they have inducible levels of CD4 allowing one to vary them in testing for macrophage tropism. Macrophage-tropic viruses are better at infecting cells with low levels of CD4. Earlier studies had indicated that macrophage tropism was the nature of the transmitted virus [2, 191] but more recent studies have suggested that macrophage tropism [10, 192, 193] and low CD4 utilization [123, 163] may not be not necessary for transmission.

## Impact of approaches used to study the transmitted virus upon discrepancies in character of the transmitted/founder (T/F) HIV-1

There have been differing findings on the character and property of the T/F and acute infection viruses, potentially obscuring our ability to identify specific properties linked to transmissibility or selection at transmission. These differing findings mainly originate from differences in the methodologies and experimental designs used in these studies although other factors like study population, transmission risk factors, lab-to-lab definition of acute and chronic virus play part as well. The first problem is that there is approximately 10 days delay between the infection by the HIV-1 virus and the detection of the viral RNA in blood that makes it difficult to monitor the actual transmission process. Depending on when the actual virus was sampled, can lead to different conclusions. The only way around this is to sample tissues on a regular basis, but this is highly invasive.

The second issue lies in the different approaches for sequencing HIV-1. In order to study selection of the transmitted virus in detail, samples are taken from the donor blood as well as from the recipient blood. This is followed by subcloning and bulk sequencing [194, 195], single genome amplification (SGA)-based analysis [6–8, 93] or deep sequencing-based analysis [196]. Earlier studies of the T/F virus relied on collecting the sample material a few months following appearance of infection symptoms followed by bulk PCR-based methods, cloning, sequencing and phylogenetic analysis [27, 105, 110, 194, 197, 198] or heteroduplex tracking assay (HTA) [194, 197, 198]. These studies reported that the transmitted virus found in the acute phase of infection is homogenous as contrasted to more heterogeneous population found in the chronic infection and that the acute virus utilizes the CCR5 coreceptor [105, 194]. These and other [1, 2] early studies laid the groundwork supporting the conclusion that HIV-1 infection involves a transmission bottleneck.

Some of these early studies used samples that were several months post-transmission and this is too late to reliably capture the properties of the initial transmitted virus and reduce the likelihood of detecting multiple T/F viruses—on at least a theoretical level, the longer the time between transmission and sampling for these studies, the higher the likelihood that some of the initial T/F will be outcompeted and disappear. There are significant limitations to each of these approaches. The bulk PCR-based approaches are compromised by introduction of *Taq* polymerase-based errors due to lack of proof reading capability of the enzyme leading to incorporation of nucleotides on the sequence leading to exaggeration of the true diversity of the viral population. In addition, *Taq* polymerase-based template switching caused by premature termination of the elongation step, products of which are used to prime the template in the subsequent PCR amplification step leading to artificial recombination in vitro. Secondly, these approaches are also faced with a problem of template resampling due to bias against or towards particular sequences, including unequal representation of the template in the sample. The template that is highly represented is the one that is usually amplified, making it difficult to detect templates represented in minor populations. It has been reported that they miss the variants that are represented below 20% in frequency [199, 200]. Further, cloning bias cannot be avoided, which results in an analysis skewed towards the variants that can be most easily cloned. The standard bulk sequencing thus does not accurately reflect viral diversity in an HIV-1 quasispecies.

SGA-based methods address some of these limitations. In SGA, RNA or cDNA is serial diluted, in order to identify the dilution at which no more than 25 or 30% of PCR reactions are positive. In such cases, the sample is sufficiently diluted that most of the resulting amplification products have been amplified from a single template [7]. When only a single template gives rise to the PCR product, artificial recombination through template switching cannot happen. Also the effect of cloning bias is strongly reduced because different sequences are not competing in the same transformation and growth reaction. However, this approach is very labor-intensive and reagent-intensive and is limited because it is expensive to generate and sequence very large numbers of clones.

Deep sequencing is the latest approach and affords the opportunity to handle a large data set that is a better representation of the viral population in the infected donor or recipient and PCR is not needed before the library preparation step avoiding the serious artifacts of this step. It offers the great opportunity of studying the viral genetic diversity in unprecedented details that earlier technologies couldn’t. Importantly, it also allows the use of primer IDs, which makes it possible to match each PCR product to its original template cDNA molecule generated in the reverse transcriptase reaction [196], and hence to calculate a consensus sequence for each cDNA used as a template for PCR, and to distinguish and account for template resampling and most PCR-based errors [196].

Finally, the choice of cohort from which to study transmitted viral isolates can lead to different results. In studying T/F virus some research groups have followed linked transmitting pairs where the samples are obtained from blood of the donor and the recipient [17, 97, 98] while others have looked at sequence data of acutely infected people and a unlinked chronically infected cohorts [11, 106, 156]. Both of these approaches have their advantages and disadvantages. One of the advantages of following discordant couples is the opportunity to capture the virus near the time of transmission but it can take a long time to gather data from such a cohort [17]. On the other hand, gathering large sets of sequences from unlinked acute and chronic viruses results in a much greater statistical power to detect differences and often allows for deeper analysis and comparisons. Because of the very high level of heterogeneity of HIV-1, it becomes important that a large sample size is used in generalizing findings.

## Conclusions

The HIV-1 transmission bottleneck is generally thought to originate primarily from the physical and immunological properties of the recipient’s healthy genital mucosa [4, 10, 12, 201, 202]. The bottleneck was originally understood as a physical barrier that would prevent most viruses from infecting the new host, irrespective of viral characteristics. However, over the last several years it became apparent that the transmitted founder virus is rarely the dominant variant in the donor, suggesting that the transmission bottleneck is not entirely stochastic but also involves the selection of specific viral phenotypes [3, 4, 17, 156]. We have reviewed here the characteristics identified to date that appear to change between chronic and T/F isolates.

Notably, an apparent similar [12, 15] though perhaps less severe [14] bottleneck can be observed in intravenous drug users (IVDU) suggesting that the transmission bottleneck does not absolutely require the physical barrier and/or defenses of the mucosal surface of the recipient. The transmission bottleneck observed in IVDU may reflect selection that does not require the presence of a mucosal surface as a barrier to infection. Perhaps these selective events operate during the establishment of the new, nascent infection. Similar selective processes may operate after a sexually transmitted virus crosses the genital tract mucosal barriers (see Fig. 1). In addition, the observation that different HIV-1 lineages can frequently be found in the genital tract as compared to the blood [16–21], suggests that the transmission bottleneck of sexual transmission may not be confined to the recipient but may also sometimes extend to selective events in the donor.
